# Determinants of Survival and Prognostic Factors in Patients Undergoing Liver Resection for Primary Hepatic Carcinoma—A Follow-Up Study

**DOI:** 10.3390/clinpract15070121

**Published:** 2025-06-26

**Authors:** Unenbat Gurbadam, Gantuya Dorj, Aryabilig Otgongerel, Munkhtsetseg Janlav, Serod Khuyagaa, Tsenguun Ganbat, Tserendorj Demchig, Amgalantuul Batdelger, Batsaikhan Bayartugs, Munkhdelger Byambaragchaa, Yerbolat Amankeldi, Munkhzaya Chogsom, Chinburen Jigjidsuren, Bayart-Uils Bayar, Lkham Nyam-Osor

**Affiliations:** 1 Hepatobiliary Pancreatic Transplantation Centre, National Cancer Centre of Mongolia, Nam Yan Ju Street, Ulaanbaatar 13370, Mongolia; unenbat@nccm.gov.mn (U.G.); pti1220363@pt.mnums.edu.mn (A.O.); tserendorj@nccm.gov.mn (T.D.); amgalantuul@nccm.gov.mn (A.B.); batsaikhan@nccm.gov.mn (B.B.); dr.munkhdelger@nccm.gov.mn (M.B.); yerbolat@nccm.gov.mn (Y.A.); mzaya-ch@cancer-center.gov.mn (M.C.); chinburen@cancer-center.gov.mn (C.J.); bayart-uils@nccm.gov.mn (B.-U.B.); lkham@nccm.gov.mn (L.N.-O.); 2 Department of Epidemiology and Biostatistics, School of Public Health, Mongolian National University of Medical Sciences, Zorig Street, Ulaanbaatar 14210, Mongolia; serod@mnums.edu.mn; 3 Department of Biochemistry, School of Biomedicine, Mongolian National University of Medical Sciences, Zorig Street, Ulaanbaatar 14210, Mongolia; 4 Institute of Medical Sciences, Mongolian National University of Medical Sciences, Zorig Street, Ulaanbaatar 14210, Mongolia; pti1220365@pt.mnums.edu.mn

**Keywords:** hepatectomy, liver cancer, vascular invasion, prognosis

## Abstract

**Background:** Mongolia has a high incidence of hepatocellular carcinoma (HCC), with 85.6 cases per 100,000 population and 70% diagnosed at an advanced stage. HCC accounts for 35% of all cancer-related deaths in the country. The primary treatment for HCC remains hepatotectomy. This study aims to investigate the factors affecting the prognosis of patients undergoing liver resection for HCC in Mongolia. **Materials and Methods:** A retrospective cohort study was conducted using data from the National Cancer Centre’s eHealth program and cancer registry. The study enrolled 1100 patients who underwent liver resection from 2015 to 2018, with a follow-up period of 5.25–9.25 years to determine survival rates. **Results:** The study included 980 patients, with a male-to-female ratio of 1.2:1 and an average age of 60 years. Tumour stage II patients had the highest survival rate (46.55%), and those with stage IIIb had the lowest (1.51%) (*p* = 0.0001). Smaller tumours (≤5 cm) were associated with better survival (*p* = 0.0006). Histologically, 19.4% had liver cirrhosis, and 80.7% had liver fibrosis. The preoperative median AFP level was 23.9 ng/mL (range 0–121,000 ng/mL). The average survival time post-liver resection was 6.675 years (*p* = 0.0006). Factors such as blood loss (*p* = 0.0004), vascular invasion (MaVI-*p* < 0.0001, MVI *p* = 0.0011), tumour size ≤ 5 cm (*p* = 0.0007), and elevated AST and ALT levels significantly influenced long-term survival (*p* = 0.0004, respectively). **Conclusions:** The study identified key prognostic factors influencing survival rates in HCC patients post-liver resection. Minimising blood loss, early detection, and managing vascular invasion, along with early-stage detection and treatment, are crucial for improving patient outcomes.

## 1. Introduction

Mongolia is an Asian country between Russia in the north and China in the south. It is a unique country with 566,460 square kilometres and a population of 3.4 million, making it the most sparsely populated country. In 2022, the GDP was USD 4242 per capita, ranking 111th in the world [1].

As of 2023, hepatocellular carcinoma (HCC) records approximately 905,677 new cases and 830,180 deaths worldwide [2]. Elevated age-standardised incidence rates (ASIR) and age-standardised mortality rates (ASMR) are mostly reported in Asian and Sub-Saharan African regions [2]. Mongolia is endemic to HCC, exhibiting the highest rates for both ASIR at 85.6 per 100,000 individuals and ASMR with 80.6 per 100,000 individuals [3]. It is reported that Mongolian HCC exhibits distinct molecular characteristics, including a high mutational load and a new mutational signature linked to genotoxic environmental factors [4].

HCC in Mongolia is primarily driven by chronic infections with hepatitis B virus (HBV) and C (HCV), as well as alcohol consumption [5,6,7,8,9,10,11,12]. Moreover, the prevalence of hepatitis D virus (HDV) among HBV-infected individuals was notably high, with studies indicating that approximately 5% of HBsAg-positive patients also test positive for anti-HDV [13].

The National Cancer Centre of Mongolia (NCCM) stands as the premier public tertiary hospital in the country, specializing in comprehensive cancer diagnosis, treatment, and management, serving as the foremost authority in oncology care nationwide.

Within the NCCM, the Hepatobiliary and Pancreatic Surgery Department excels in the specialized treatment of cancers affecting the liver, biliary tract, and pancreas. The department follows clinical guidelines [14] based on the North American National Comprehensive Cancer Network (NCCN) [15] for diagnosing and managing HCC. Their comprehensive treatment modalities include hepatotectomy, liver transplantation, and advanced systemic therapies such as targeted therapy and immunotherapy for advanced stages of HCC [16].

NCCM hosts the National Cancer Registry of Mongolia (NCRM), a hospital-based registry to collect data on newly registered cancers, deaths from cancer, and cancer care nationwide [10,17,18]. While a body of work investigated the survival outcomes in patients post-surgery [19,20,21], only limited information is available from HCC-endemic countries [7,22,23]. This large retrospective cohort is needed to provide comprehensive data and enhance our understanding of survival outcomes in these regions. We hypothesized that the survival rates and prognostic factors in HCC patients who underwent surgical treatment in Mongolia are significantly influenced by chronic hepatitis infections (HBV, HCV, HDV), alcohol consumption, and other risk factors. This study aims to provide comprehensive insights into patient outcomes and the impact of these risk factors in an HCC-endemic country.

## 2. Materials and Methods

A hospital-based retrospective study using the NCRM registry was employed to investigate the prognosis and survival rates. There were 1100 patients diagnosed with HCC who underwent liver resection from January 2015 to December 2018. Patients diagnosed with bile duct cancer, concurrent organ cancers, uncertain pathological cases, benign liver tumours, primary cancer metastases, and those who had undergone prior treatments were excluded from the study, resulting in a total exclusion of 30 cases. All diagnoses of HCC were diagnosed per radiology and confirmed by histopathological tests. The Child–Pugh score was used to evaluate the severity of chronic liver disease and cirrhosis, incorporating factors such as bilirubin levels, serum albumin, INR, ascites, and hepatic encephalopathy. Before surgery, each patient underwent biochemical analyses, and alpha-fetoprotein (AFP) and viral hepatitis testing. According to national guidelines, gamma-glutamyl transferase (GGT) and alkaline phosphatase (ALP) tests are performed only on patients who require them [14]. In this study, no patients were required to be tested for GGT or ALP.

Imaging techniques such as abdominal ultrasonography, CT, and MRI were used to assess tumour resectability.

In our hospital, surgeries are classified using Couinaud’s system, which divides the liver into eight segments, each with its own vascular supply and drainage [24]. This system aids in precise surgical planning and execution [14].

Macrovascular Invasion (MaVI): This refers to the invasion of tumour cells into larger vessels, including major vessels and branches of the portal vein. Vessels involved in MaVI are typically greater than one mm in diameter.

Microvascular Invasion (MVI): This is defined as the presence of tumour cells within small vessels, specifically those with a diameter of less than one mm. This assessment is conducted independently by both the surgeon and the pathologist. MVA encompasses only small vessels.

Major Liver Resection: This is defined as the removal of four or more segments of the liver.

Minimal Liver Resection: This involves the removal of fewer than four segments of the liver, which usually denotes MVI [24]. Furthermore, the presence of MVI (<1 mm or positive) is assessed by both the surgeon and the pathologist at our hospital.

Patients were operated on with radical intent, following the reported guidelines [14]. R0 and R1 classifications were determined based on histological examination in all patients who underwent macroscopic resection of the entire tumour mass. Solitary fibrous tumours are rare growths that can develop in various parts of the body. These tumours originate from connective tissues, which support other tissues in the body [25].

The Ishak classification is a system used to assess liver fibrosis and inflammation in chronic hepatitis.

Prognostic factors included in the data analysis were sex, age, HBV, HCV, HDV, alcohol consumption, aspartate aminotransferase (AST), alanine aminotransferase (ALT), serum AFP, cirrhosis, the extent of liver resection, blood loss, number and size of tumours, type of operation (minor or major), invasiveness of the resection margin, American Joint Committee on Cancer (AJCC) Tumour Nodes Metastasis (TNM) stage, and vascular invasion.

From the patients’ records, we collected information on follow-up, which was conducted in the outpatient department of NCCM every 3 months for the first 2 years, with a physical examination, liver function tests, levels of AFP, chest radiography, and abdominal ultrasonography. Every 6 months, the patients underwent a CT scan and/or MRI. The recurrence of HCC was evaluated with abdominal ultrasonography or CT and hepatic artery angiography.

In the study, survival outcomes are presented as overall survival rates. Overall survival refers to the proportion of patients who were still alive after the treatment, regardless of whether the disease has recurred. The last date of study follow-up was 1 April 2024, resulting between 5.25 and 9.25 years

## 3. Statistical Analysis

In the preliminary data analyses, all selected variables were analysed using means for continuous variables and proportions for categorical ones. We evaluated overall survival rates in patients who had undergone resection and were discharged from the hospital, tracking from the surgery date to recurrence (if any), study end, or death. In patients with HCC recurrence, data were censored at the time of diagnosis of recurrence.

Variables were selected based on their potential link to recurrence, guided by literature and our clinical expertise. Kaplan–Meier survival curves were developed for these variables and hazard ratios for each variable were calculated. Analysis was conducted using univariate methods, focusing on the individual effects of each variable. Cases with incomplete data were excluded from the analysis. All statistical analyses were performed using SPSS 28, with a *p*-value of less than 0.05 considered statistically significant.

## 4. Independent Variables Included

Demographic factors: age, sex.

Clinical factors: active alcohol consumption ranging from moderate to excessive, AST, ALT, AFP levels, cirrhosis, HBV, HCV, HDV.

Surgical factors: extent of liver resection, blood loss, number and size of tumours, type of operation, invasiveness of resection margin.

Pathological factors: AJCC TNM stage, vascular invasion.

Dependent variables included (i) prognosis (survival rates), (ii) clinical outcomes.

## 5. Ethical Approval

The study protocol was reviewed and approved by the Research Ethics Committee, Mongolian National University of Medical Sciences (2023/3-08).

## 6. Results

**Figure 1:**
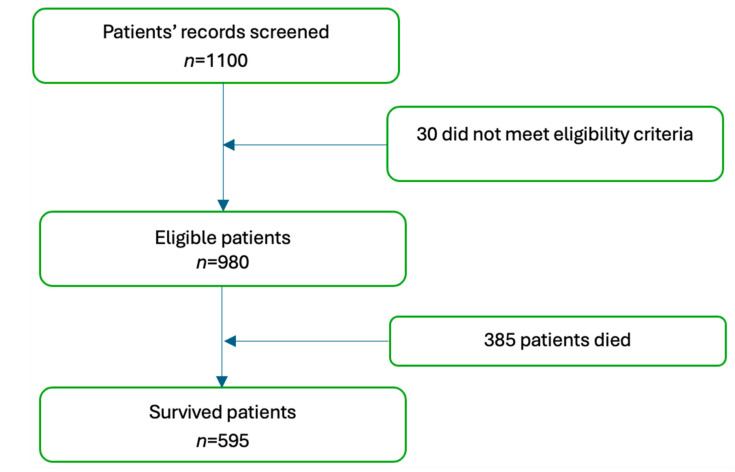
Study participants flowchart.

Between January 2015 and December 2018, data from 980 eligible patients were included in the final analysis. The proportion of male patients was slightly greater (54.8%) than the female counterparts (45.2%). Among newly diagnosed patients, comorbidities included gastrointestinal disease (27.04%), hypertension (15.31%), diabetes (3.16%), and urinary diseases (1.22%) (Table 1). At a median follow-up of 7.5 years (5–9), 385 patients died, 595 patients survived (Figure 1), and 308 patients experienced cancer recurrence.

The cohort’s actuarial overall survival (OS) was 60.71% by the end of the follow-up period. Recurrence was diagnosed at a median of one year after surgery, and the actuarial recurrence-free survival (RFS) was 68.58%. Out of patients with recurrent cancer, 294 underwent angiography transarterial chemoembolisation (TACE)/radiofrequency ablation (RFA). By the end of study, a total of 595 patients had survived.

The median age of patients was 60 (17–85). The average tumour size was 4.4 cm, with major resections up to 5 cm and minor resections at up to 4 cm (*p* = 0.0001) (Table 1). The median of large tumour diameter was 22 cm (1–22), and multiple tumours were present in 15.4% of cases. Major vascular invasion was identified in 62 patients (6.3%), whereas small vascular invasion was present in most patients (60.1%). The preoperative median of AFP was 23.9 ng/mL (range 0–121,000 ng/mL) (Table 1). Viral hepatitis was detected in 74.28% of patients, most being HCV infection (*n* = 353, 36.02%).

Preoperative tumour size assessment revealed that 65.3% (*n* = 640) of tumours were ≤5 cm, while 34.6% (*n* = 340) were >5 cm. When applying the ISHAK classification, it was observed that almost one in five patients were diagnosed with liver cirrhosis (19.29%), indicating advanced liver scarring. In contrast, the remaining 80.71% of the patients exhibited varying degrees of liver fibrosis, reflecting different stages of liver tissue damage but not reaching the threshold of cirrhosis (Table 1).

**Figure 2:**
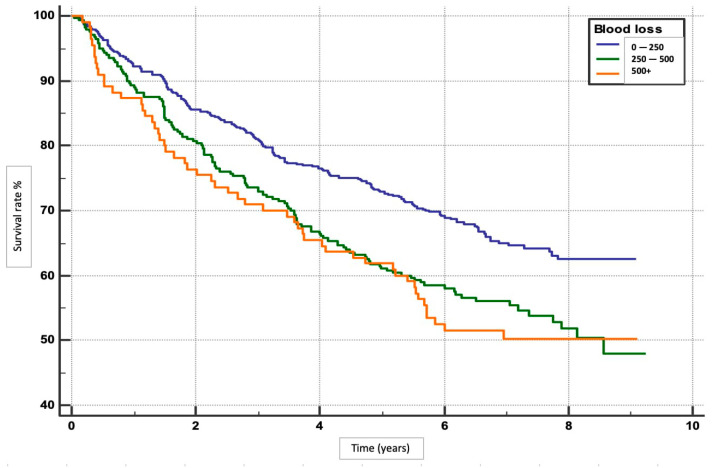
Post-surgical survival in relation to blood loss.

**Figure 3:**
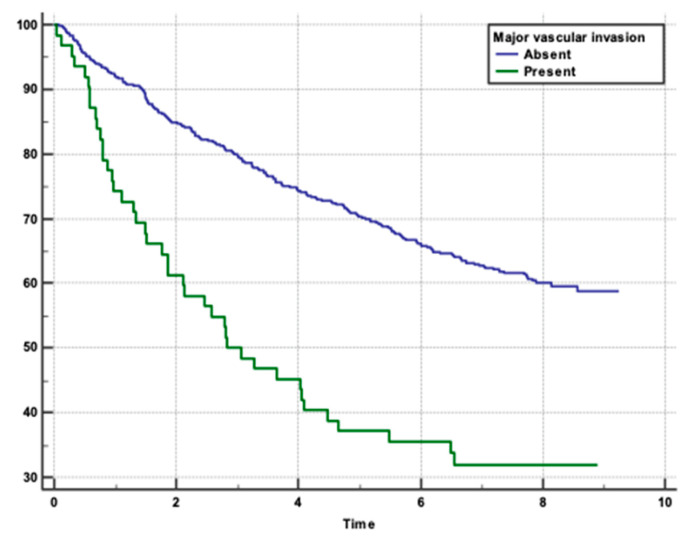
Post-surgical survival in relation to MaVI.

**Figure 4:**
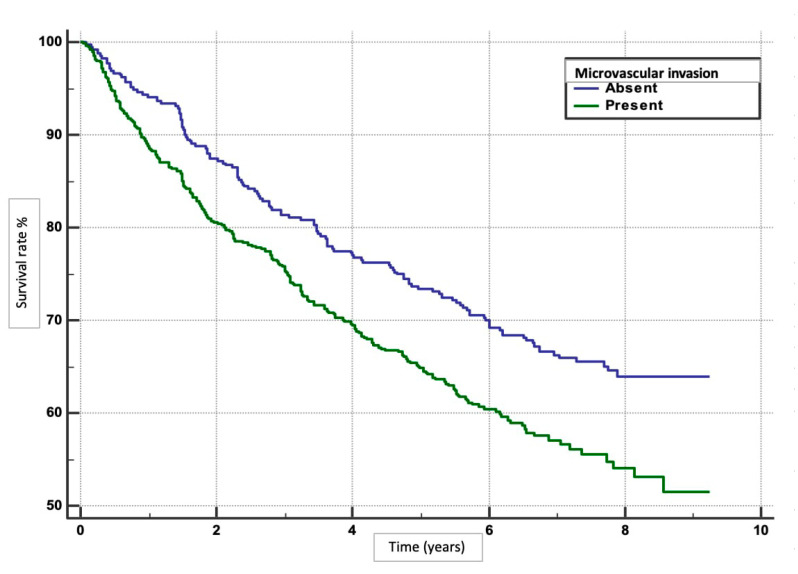
Post-surgical survival in relation to MVI.

**Figure 5:**
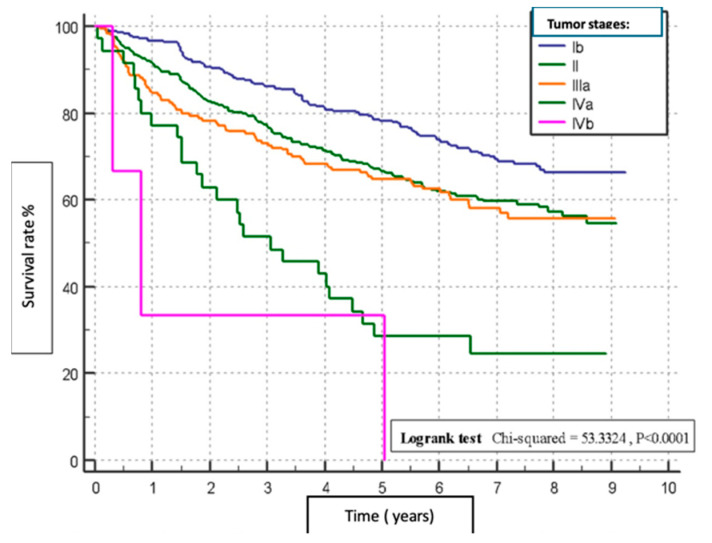
Comparative analysis of post-surgical survival based on tumour stage.

**Figure 6:**
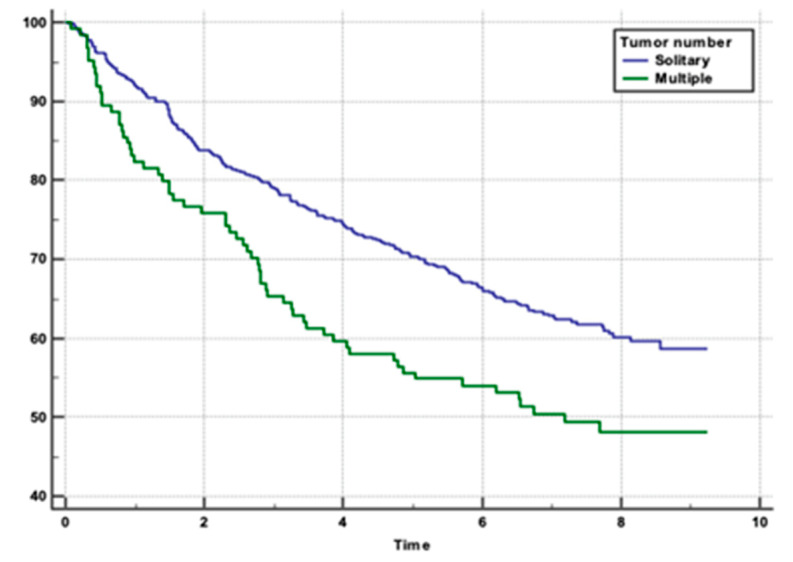
Post-surgical survival in relation to tumour number.

We performed an analysis to determine the risk factors for overall survival rates in patients with HCC following hepatotectomy. Patients with minimal blood loss (0–250 mL) had the highest survival rate (*p* = 0.0004), (Figure 1). Absence of MaVI and MVI correlated with higher survival rates (*p* < 0.0001, *p* = 0.0011), respectively (Figure 2 and Figure 3). Tumour stage II patients had the highest survival rate (46.55%), and those with stage IIIb had the lowest (1.51%) (*p* = 0.0001) (Figure 4). Smaller tumours (≤5 cm) and tumour numbers (solitary vs multiple) were associated with better survival (*p* = 0.0006) (Figure 5 and Figure 6).

In the study, several factors were found to be independently associated with the survival outcome following hepatectomy. These factors included volume of blood loss, presence of MaVI and MVI, size and number of tumours, and levels of ALT and AST. The results indicate that higher volumes of blood loss were associated with poorer survival outcomes, with HR of 1.4535 for 250–500 mL and 1.5831 for >500 mL compared to 0–250 mL. The presence of MaVI and MVI significantly increased the risk of mortality, with HRs of 2.5398 and 1.4145, respectively. Patients with multiple tumours had a higher risk of death compared to those with a solitary tumour (HR 1.4978), and larger tumours (>5 cm) were associated with worse survival compared to smaller tumours (≤5 cm; HR 1.4325). Elevated levels of ALT and AST were also linked to poorer survival outcomes. For ALT, HRs were 1.5747 for 50–100 and 1.4173 for >100 compared to 0–50. Similarly, for AST, HRs were 1.2985 for 50–100 and 1.6576 for >100 compared to 0–50 (Table 2 and Table 3).

## 7. Discussion

To the best of our knowledge, this is the first study investigating survival outcomes and prognostic factors in patients with HCC undergoing hepatectomy in Mongolia, a country with one of the highest burdens of HCC. In the study, the median survival time for patients who underwent liver resection was 6.675 years. Post-surgery, cancer recurred in about one-third of the patients (*n* = 308), and 12 patients with recurrence died. By the end of the study period, survival rate in patients receiving hepatectomy was 60.71%. The findings highlight several critical factors influencing patient outcomes, particularly focusing on blood loss, vascular invasion, tumour characteristics, and liver function markers.

### 7.1. Blood Loss and Survival Outcomes

The volume of intraoperative blood loss was found to be a critical determinant of survival outcomes. In our study, patients experiencing greater blood loss (250–500 mL and >500 mL) were at up to 58% higher risk of dying compared to those with minimal blood loss (0–250 mL). This finding aligns with previous studies that have highlighted the importance of minimizing blood loss during hepatectomy to improve postoperative outcomes [26]. A US study analysed 1803 consecutive hepatic resection cases over 10 years [27]. In this study, the median blood loss was 600 mL and 49% of patients required transfusions. Blood loss significantly predicted survival outcomes, raising perioperative mortality risk by 69% [27].

Minimizing intraoperative blood loss is vital to reduce hypovolemic shock and improve outcomes. National guidelines on hepatectomy indicate preoperative planning, stopping anticoagulants 48 h before surgery, using cell salvage, administering tranexamic acid, and employing proper patient positioning and regional anaesthesia [14].

### 7.2. Vascular Invasion

Literature findings demonstrate that the presence of vascular invasions is associated with decreased survival.

MVI: Recognizing MVI helps in stratifying patients’ risk and tailoring postoperative therapies. Early detection of MVI can lead to timely interventions, potentially improving survival rates. In our study, the absence of MVI correlated with better outcomes (HR 1.4145) than in those with MVI. A systematic review analysed data from 37 studies enrolling 14,096 patients and reported the impact of postoperative complications on long-term survival outcomes [28]. The HR for overall survival in patients with MVI was 1.34 (95% CI: 1.19–1.51), indicating that the presence of MVI increases the risk of mortality by 34% compared to those without MVI [28]. The effect of MVI on the post-operative long-term prognosis was reported in a subsequent systematic review and meta-analysis of 14 eligible studies with 3033 patients [29]. This review focused on studies reporting solitary small HCCs with maximum tumour diameters of up to 2 cm, 3 cm, or 5 cm [29]. Similar to our findings, a meta-analysis found that the presence of MVI was associated with significantly worse overall survival (HR 2.42, 95% CI: 1.96–2.99, *p* < 0.001) [29].

On the other hand, some findings demonstrate that MVI has robust predictive value for survival in HCC patients following surgery. A study by Zhang et al. analysed data from 300 patients with HCC, using Kaplan–Meier survival curves and Cox proportional hazards models to compare the prognostic significance of MVI and tumour size. They found that tumour size was a significant predictor of survival (HR 2.5, 95% CI 1.8–3.5, *p* < 0.001), while MVI was not independently significant (HR 1.2, 95% CI 0.9–1.6, *p* = 0.15). The study validated these findings using internal and external patient cohorts and statistical techniques, confirming that tumour size, rather than MVI, better predicts survival outcomes in HCC patients [30]. This suggests that while MVI may indicate aggressive tumour characteristics, it does not independently affect overall survival rates as strongly as tumour size does [30].

Identifying MaVI is essential for prognosis and treatment planning. Recommended approaches for patients with MaVI include aggressive treatment strategies such as advanced systemic therapies (e.g., targeted therapy, immunotherapy) and close monitoring for disease progression. In our study, patients without MaVI had a significantly higher survival rate compared to those with MaVI, as indicated by a HR of 2.5398. This finding underscores the critical impact of vascular invasion on the prognosis of patients with HCC. The presence of tumour thrombi in major blood vessels, such as the portal vein, is associated with a poor prognosis and high recurrence rates [31,32]. Vascular invasion facilitates the dissemination of cancer cells, leading to metastasis and recurrence, which significantly compromises patient outcomes. Our results align with existing literature, which consistently demonstrates that major vascular invasion is a key determinant of survival in HCC patients [33]. A study was conducted at Taipei Veterans General Hospital in Taiwan, enrolling 2654 patients. About one-third of the patients had MaVI. The study employed Cox proportional hazards models to identify risk factors associated with decreased survival in HCC patients. Among the significant predictors identified in the study, the presence of MaVI was associated with decreased survival. The HR for MaVI was 1.599 in the curative treatment group and 1.804 in the noncurative group. This indicates that MaVI significantly increases the risk of mortality in both groups [34].

Our findings suggest that treatment plans for HCC should prioritize the assessment of vascular invasion. Patients without MaVI may benefit from more aggressive surgical approaches and adjuvant therapies, given their relatively better prognosis. Conversely, for patients with MaVI, a multidisciplinary approach that includes systemic therapies, such as tyrosine kinase inhibitors (TKIs) and immune checkpoint inhibitors (ICIs), in combination with locoregional treatments, may be recommended [31,32].

### 7.3. Tumour Characteristics

Tumour Size/Number: Accurate assessment of tumour size and number is vital for staging and determining the appropriate surgical approach. Larger or multiple tumours may necessitate more extensive resections and adjuvant therapies. The results of the present study indicated that tumour size and number were also significant predictors of survival. Smaller tumours (≤5 cm) were associated with better survival outcomes compared to larger tumours (>5 cm), with an HR of 1.4325. Additionally, patients with solitary tumours had better survival rates than those with multiple tumours (HR 1.4978). Multiple tumours often indicate a more advanced stage of disease and a higher likelihood of intrahepatic metastasis, which complicates treatment and reduces the chances of achieving complete remission.

Tumour size is one of the first five significant predictors of survival in HCC [30,35,36,37]. The systematic review analysed 72 studies involving 23,968 patients with HCC. It identified tumour size as one of the most robust predictors of death, with larger tumours significantly associated with poorer survival outcomes (HR 1.8, 95% CI 1.5–2.2, *p* < 0.001) [37]. This can be attributed to smaller tumours being less likely to have invaded major blood vessels or metastasized, making them more amenable to curative treatments such as surgical resection or local ablation.

### 7.4. Liver Function Markers

Monitoring liver enzymes and biomarkers is crucial for assessing liver function and predicting surgical outcomes in HCC patients undergoing resection. In our study, elevated levels of ALT and AST were linked to poorer survival outcomes. For ALT, the HRs were 1.5747 for 50–100 U/L and 1.4173 for >100 U/L compared to the 0–50 U/L group. Similarly, for AST, the HRs were 1.2985 for 50–100 U/L and 1.6576 for >100 U/L. Similar findings can be found in the literature [38,39]. One study involved 601 Chinese patients with HCC who underwent hepatectomy [38]. It used retrospective analysis to identify prognostic factors affecting overall survival. Key findings indicated that elevated preoperative ALT levels (>40 U/L) and AST levels (>35 U/L) were significantly associated with poorer overall survival (HR 1.38, 95% CI 1.10–1.73, *p* = 0.005) [38].

Monitoring ALT and AST levels provides insights into liver function and potential liver damage. Elevated levels can indicate underlying liver conditions, guiding the need for further diagnostic evaluations and management strategies.

## 8. Age and Sex

In our study, age and sex were not significantly associated with survival outcomes post hepatectomy. The impact of age and sex on survival rates following hepatectomy is debatable in the literature. A study analysed 1547 HCC patients from two European cohorts (University Hospital of Bern, Switzerland, and General Hospital Vienna, Austria). Using Kaplan–Meier curves and Cox regression models, it found no significant difference in OS between males and females (HR = 1.02, 95% CI: 0.89–1.17, *p* = 0.76). Age influenced OS, particularly for those aged 60–65 (HR = 1.25, 95% CI: 1.05–1.48, *p* = 0.01), but after adjusting for treatment factors, the effect of age on survival was no longer significant (HR = 1.10, 95% CI: 0.92–1.31, *p* = 0.30) [40]. Nevola et al. provided a comprehensive review of existing literature and clinical data to analyse the influence of sex on HCC development and outcomes [41]. Key findings indicated that men have a higher incidence of HCC and tend to develop it at a younger age compared to women. The study highlighted that male sex was associated with a higher risk of aggressive HCC (HR = 1.35, 95% CI: 1.10–1.65, *p* < 0.01), while female patients had better overall survival rates (HR = 0.85, 95% CI: 0.75–0.97, *p* = 0.02) [41]. These findings suggest that while age and sex may not always significantly impact survival outcomes post-hepatectomy, patient characteristics, including comorbidities and lifestyle factors, play a crucial role in influencing survival and should be carefully considered in clinical decision-making.

Addressing these factors comprehensively can enhance patient outcomes and optimize the management of HCC in Mongolia.

## 9. Limitations

Despite our efforts, this study is not without limitations. The retrospective design may introduce selection bias and limit our ability to establish causality. Additionally, data from a single centre in Mongolia may not be generalizable to other countries. However, it is important to note that the National Cancer Centre of Mongolia is the sole institution providing comprehensive cancer care in the country. Furthermore, we utilised the largest and single hospital-based data encompassing all registered cancers, cancer-related deaths, and cancer care, which enhances the robustness of our findings from the HCC-endemic country.

## 10. Conclusions

Our findings underscore the importance of preoperative optimization, surgical techniques to minimize blood loss, and vigilant follow-up for high-risk patients in shaping clinical practice in Mongolia. These strategies can significantly improve patient outcomes and serve as a model for other HCC-endemic regions. Future directions include prospective studies and broader multicentre collaborations to validate and expand upon our results, ultimately enhancing the global management of hepatocellular carcinoma.

## Figures and Tables

**Table 1:** Demographic and clinical variables of patients undergoing liver resection surgery (*n* = 980).

Variable	Category	Number of Cases, *n*	%
Age	≤25	4	0.20
	26–45	50	9.59
	46–60	442	39.59
	61+	484	50.61
Sex	Male	537	54.80
	Female	443	45.20
Family history	Yes	79	8.06
	No	894	91.22
	I don’t know	7	0.71
Comorbidities	Diabetes	31	3.16
	Hypertension	150	15.31
	Gastrointestinal disease	265	27.04
	Urinary disease	12	1.22
ISHAK	Non cirrhosis	791	80.71
	Cirrhosis	189	19.29
Tumour number	Solitary	681	69.49
	Multiple	299	30.51
Surgical margin (R0/R1)	Negative	976	99.59
	Positive	4	0.41
Macrovascular invasion (MaVI)	Absent	918	93.67
	Present	62	6.33
Microvascular invasion (MVI)	Absent	391	39.90
	Present	589	60.10
Viral hepatitis	HBV	293	29.90
	HCV	353	36.02
	HBV + HCV	14	1.43
	HBV + HDV	68	6.94
Alfafetoprotein (AFP)	0–6.05	234	23.88
	>6.05	746	76.12
Total bilirubin (TBIL)	0–17.1	739	75.41
	>17.1	241	24.59
Albumin (ALB)	<35	142	14.49
	35–51	833	85.00
	>51	5	0.51
International Normalised Ratio (INR)	<0.9	23	2.35
	0.9–1.1	511	52.14
	>1.1	446	45.51

**Table 2:** Univariate analysis of factors associated with patient overall survival (*n* = 595).

Variable	Category	Overall Survival		*p* Value
		*n*	%	
Blood loss	0–250 mL	387	94.62	0.0004
	250–500 mL	152	37.16	
	>500 mL	56	13.69	
MaVI	Absent	575	96.64	<0.0001
	Present	20	3.36	
MVI	Absent	256	43.03	0.0011
	Present	339	56.97	
Tumour stage	Ib	192	32.61	0.0001
	II	280	46.55	
	IIIa	114	19.33	
	IIIb	9	1.51	
Tumour size	≤5 cm	387	66.96	0.0006
	>5 cm	191	33.04	
Tumour number	Solitary	425	71.43	0.0038
	Multiple	61	10.25	
ISHAK	Cirrhosis	478	80.34	0.5826
	Non–cirrhosis	117	19.66	
ALT	0–50 U/L	292	48.99	0.0004
	51–100 U/L	169	28.36	
	100+ U/L	135	22.65	
AST	0–50 U/L	326	54.79	0.0004
	51–100 U/L	186	31.26	
	>100 U/L	83	13.95	

**Table 3:** Independent factors associated with patient overall survival (*n* = 595).

Factor	Comparison	Hazard Ratio (HR)	95% CI of HR
Blood loss	0–250 mL vs. 250–500 mL	1.4535	1.1537 to 1.8314
	0–250 mL vs. >500 mL	1.5831	1.1339 to 2.2103
MaVI	absent vs. present	2.5398	0.1470 to 0.3818 2.6191–6.8040
MVI	absent vs. present	1.4145	0.5811 to 0.8726 1.1460–1.7210
Tumour number	solitary vs. multiple	1.4978	1.1623 to 2.1845
Tumour size	≤5 cm vs. >5 cm	1.4325	1.1759 to 1.8151
ALT	0–50 vs. 50–100 U/L	1.5747	1.2475 to 1.9876
	0–50 vs. 100+ U/L	1.4173	1.0987 to 1.8283
AST	0–50 vs. 50–100 U/L	1.2985	1.0362 to 1.6271
	0–50 vs. >100 U/L	1.6576	1.2513 to 2.1959

## Data Availability

The raw data supporting the conclusions of this article will be made available by the authors on request.

## Abbreviations

AFP	Alpha-fetoprotein.
AJCC	American Joint Committee on Cancer.
ALT	Alanine aminotransferase.
ASIR	Age-standardised incidence rates.
ASMR	Age-standardised mortality rates.
AST	Aspartate aminotransferase.
HCC	Hepatocellular carcinoma.
HBV	Hepatitis B virus.
HCV	Hepatitis C virus.
HDV	Hepatitis D virus.
HR	Hazard Ratio.
MaVI	Macrovascular invasion.
MVI	Microvascular invasion.
NCCM	National Cancer Centre of Mongolia.
NCCN	National Comprehensive Cancer Network.
NCRM	National Cancer Registry of Mongolia.
OS	Overall survival.
PIVKA-II	Protein induced by vitamin K absence or antagonist-II.
TNM	Tumour nodes metastasis.
